# A BAC-Based Physical Map of Zhikong Scallop (*Chlamys farreri* Jones et Preston)

**DOI:** 10.1371/journal.pone.0027612

**Published:** 2011-11-16

**Authors:** Xiaojun Zhang, Cui Zhao, Chao Huang, Hu Duan, Pin Huan, Chengzhang Liu, Xiuying Zhang, Yang Zhang, Fuhua Li, Hong-Bin Zhang, Jianhai Xiang

**Affiliations:** 1 Key Laboratory of Experimental Marine Biology, Institute of Oceanology, Chinese Academy of Sciences, Qingdao, China; 2 Graduate University of the Chinese Academy of Sciences, Beijing, China; 3 Department of Soil and Crop Sciences, Texas A&M University, College Station, Texas, United States of America; Nanjing Forestry University, China

## Abstract

Zhikong scallop (*Chlamys farreri*) is one of the most economically important aquaculture species in China. Physical maps are crucial tools for genome sequencing, gene mapping and cloning, genetic improvement and selective breeding. In this study, we have developed a genome-wide, BAC-based physical map for the species. A total of 81,408 clones from two BAC libraries of the scallop were fingerprinted using an ABI 3130xl Genetic Analyzer and a fingerprinting kit developed in our laboratory. After data processing, 63,641 (∼5.8× genome coverage) fingerprints were validated and used in the physical map assembly. A total of 3,696 contigs were assembled for the physical map. Each contig contained an average of 10.0 clones, with an average physical size of 490 kb. The combined total physical size of all contigs was 1.81 Gb, equivalent to approximately 1.5 fold of the scallop haploid genome. A total of 10,587 BAC end sequences (BESs) and 167 markers were integrated into the physical map. We evaluated the physical map by overgo hybridization, BAC-FISH (fluorescence *in situ* hybridization), contig BAC pool screening and source BAC library screening. The results have provided evidence of the high reliability of the contig physical map. This is the first physical map in mollusc; therefore, it provides an important platform for advanced research of genomics and genetics, and mapping of genes and QTL of economical importance, thus facilitating the genetic improvement and selective breeding of the scallop and other marine molluscs.

## Introduction

Zhikong scallop (*Chlamys farreri* Jones et Preston) has a wide distribution along the coasts of North China, Korea, Japan, and Eastern Russia. It is a most dominant scallop species for aquaculture, and its production has reached approximately 80% of the total scallop production in China [1]. However, the frequent mass mortality of the species has seriously affected the development of its industry since 1996 [2]. To develop new scallop genotypes to manage the problem, it is necessary to have a better understanding of the molecular mechanisms underlying its economically important quantitative and qualitative traits, such as disease resistance and growth rate. Therefore, many investigations have recently focused on genome research of the species.

*Chlamys farreri* has 2*n* = 38 chromosomes and its haploid genome size is approximately 1.24 Gb [3], [4]. Significant progress has been made recently in its genomics, including development of genetic linkage maps [5]–[7], construction of large-insert genomic DNA libraries [1], [3], mapping of economically important quantitative trait loci (QTL) [7], and large-scale sequencing of expressed sequence tags (ESTs) [8]. However, there have not been a genome-wide physical map and extensive long-range genome sequence that are crucial to advanced studies of genomics available in scallop. The shortage of such genomics tools and infrastructure in the species has not only limited the molecular cloning and analysis of its genes and QTL, but also prevented the flow of genomic information from the model species and other mollusc such as oyster (*Crassostrea gigas*) to scallop. Furthermore, it is desirable to have a genome-wide physical map to sequence and assemble the scallop genome using the next-generation sequencing (NGS) technology. Therefore, a comprehensive physical map is needed for advanced research of the scallop genome.

Physical maps are crucial tools for genome sequencing, gene mapping and cloning, genetic improvement and selective breeding. Although the development of whole-genome shotgun sequence draft maps based on NGS has become cost-effective recently, a physical map remains an important component of genome sequencing projects [9]. For the large regions of repeat sequences and highly heterozygous genomes, sequencing and assembly will not be easily addressed by NGS alone. Additional tools are needed to provide anchor points to link sequence contigs and bridge the large repeat regions [10]. Physical maps, specially the contig physical maps constructed based on restriction fragment fingerprints of bacterial artificial chromosome (BAC) clones, can provide these anchor points, such as BAC end sequences (BESs) and markers, for genome sequence assembling. Moreover, genome-wide integrative physical and genetic mapping is a most efficient and economical approach to fine mapping and positional cloning of genes controlling many phenotypic traits such as QTL.

BAC contig-based physical maps have been constructed in several aquaculture species, including Nile tilapia [11], Atlantic salmon [12], catfish [13], [14], rainbow trout [15] and Asian seabass [16]. These physical maps have greatly enhanced genome research in the species. Based on the physical maps, integrated genome maps have been developed for the Atlantic salmon [17] and rainbow trout [18]. These genome resources have been proven very useful for the identification of genomic regions containing genes of economically important traits and whole genome sequencing. However, no genome-wide physical map has been reported for a species of mollusca, the largest marine organism phylum containing approximately 85,000 recognized species (http://en.wikipedia.org/wiki/Mollusca).

We have previously constructed two BAC libraries from the nuclear DNA of *C. farreri*, and identified the BACs containing six genes involved in the innate immune system of mollusc [1]. Using the BACs as a tool, we have characterized two of the six genes and mapped them to *C. farreri* chromosomes using the fluorescent *in situ* hybridization (FISH) technology [19]–[21]. In this study, we have developed a genome-wide physical map of Zhikong scallop from the BAC libraries, thus providing useful and friendly-used tools for advanced studies of its genomics, particularly whole genome sequencing and map-based cloning of economical genes and QTL in the species.

## Methods

### BAC fingerprinting

The clones were from the two previously constructed BAC libraries, Scallop-CBE and Scallop-CME [1], all 81,408 clones were used in fingerprinting. BAC clones were inoculated in 96-deep well plates, with each well containing 1.0 ml YENB medium (China patent, ZL200610046257.6) with 12.5 µg/ml chloramphenicol, from the 384-well microtiter plates of the libraries using a 96-pin replicator (BOEKEL, Feasterville, PA, USA). Therefore, each 384-well microtiter plate of BAC clones was inoculated into four 96-deep well plates as previously described [22]. The 96-deep well plates were covered with air-permeable seals (Excel Scientific, Wrightwood, CA, USA) and incubated in an environmental shaker (Thermo Scientific, Waltham, MA, USA) at 320 rpm, 37°C for 20–22 h.

The plates were centrifuged at 3,250 rpm for 10 min in a bench-top centrifuge (Eppendorf, Hamburg, Germany) to harvest the bacteria. BAC DNA was isolated using a modified alkaline lysis method [23], dissolved in 10 µl TE containing 16 µg/ml RNase (Ambion, Austin, TX, USA) in 96-microtube plates, and stored at −80°C before use.

To select restriction enzymes that are optimal for fingerprinting the BAC libraries, we tested twelve combinations of *Bam*H I, *Eco*R I, *Hin*d III, *Xba* I, *Xho* I, and *Hae* III (New England Biolabs, Ipswich, MA, USA) using 32 BACs randomly selected from the Scallop-CBE library. After comparing the fingerprint patterns of the clones and the number of bands of each clone generated on the ABI 3130xl Genetic Analyzer (Applied Biosystems, Foster City, CA, USA), the enzyme combination of *Hin*d III/*Xba* I/*Xho* I/*Hae* III was selected for generation of BAC fingerprints for the scallop genome physical mapping.

Each BAC fingerprinting reaction consisted of 10 µl of DNA (∼200 ng), 1.3 µl of 10× reaction buffer 2 (New England Biolabs), 0.13 µl of 10 mg/ml BSA, 0.07 µl of 20 U/µl *Hin*d III, 0.07 µl of 20 U/µl *Xba* I, 0.07 µl of 20 U/µl *Xho* I, 0.14 µl of 10 U/µl *Hae* III, and 1.22 µl of ddH_2_O in a total reaction volume of 13 µl. The reaction was incubated at 37°C for 2 h, and then cooled on ice and centrifuged briefly. To each reaction, a mixture containing the following was added: 2 µl of 10× PCR buffer, 1.6 µl of 25 mmol/L MgCl_2_, 0.05 µl of dNTP (100 µmol/L dATP, 100 µmol/L dCTP, 100 µmol/L dGTP, 12.5 µmol/L Gold525- dUTP (Enzo Life Science, Inc. Farmingdale, NY, USA), 0.2 µl of 5 U/µl Taq DNA Polymerase (Promega, Shanghai, China) and 3.15 µl of ddH_2_O. The reaction was continuously incubated 65°C for 40 min. The DNA were pelleted, washed, dried and dissolved in a mixture of 9.8 µl of Hi-Di formamide and 0.2 µl of the internal GeneScan-500 Rox size standard (Applied Biosystems, Foster City, CA, USA). The DNA was denatured at 95°C for 3 min, cooled on ice and then subjected to fragment analysis on the ABI 3130xl Genetic Analyzer (Applied Biosystems, Foster City, CA, USA) using POP7 polymer and 16-channels, 36-cm capillary array.

### Data processing

The fragment sizes of each BAC fingerprint profile were collected by the ABI Data Collection program. All the data collected from the ABI 3130xl Genetic Analyzer were processed using the software package FPminer [http://www.bioinforsoft.com] and GenoProfiler [http://wheat.pw.usda.gov/PhysicalMapping/]. Briefly, fragment sizes were called using an automatic algorithm in the FPminer program and exported into “bands” files. The clone-empty lane was removed, the fingerprints with fewer than 10 fragments or more than 250 fragments were removed, only the fragments between 35 and 500 bases were kept, and the background fragments were identified and removed using the FPminer embed algorithm. The data were then transferred to Genoprofiler to remove the vector fragments and unusually frequent fragments.

### BAC contig assembly and manual editing

The program FPC version 9.3 [http://www.agcol.arizona.edu/software/fpc/] was used to assemble the processed BAC fingerprint data into automatic contigs. A series of tests were conducted in which the fingerprints of overlapping clones were compared using different tolerances (from 1 to 7) and cutoffs (1e-6 to 1e-22). On the basis of these tests, a fixed tolerance of 5 and a primary cutoff of 1e-12 (1×10^-12^) were selected and used for contig assembling.

After automatic contigs assembly under the above parameters, every contig was edited to ensure that they were accurate. Then, the contigs were merged manually if their terminal clones shared 10 or more bands and their overall fingerprint patterns supported joining, followed by manual end-to-single merging at lower stringencies and processing with Dqer function.

### Physical map quality assessment

We assessed the reliability of the scallop contig physical map using several methods, including contig clone fingerprinting using new fingerprinting kits and contig reassembly, BAC-FISH, library screening, and contig BAC pool screening. First, we selected all 190 BAC clones constituting 18 contigs randomly selected from the physical map, fingerprinted them with a new four-enzyme combination (*Bam*H I/*Eco*R I/*Xho* I/*Hae* III), assembled contigs from the clones and compared with the original contigs. Second, we further analyzed the 38 positive BAC clones identified by source BAC libraries screening using the overgo probes of six genes involved in the innate immune system of molluscs in our previous study [1]. If the physical map contigs were assembled properly, the positive clones of each of the genes, if single-copy, should be assembled into a single contig. Furthermore, we also physically mapped the six positive clones of the *lgbp* gene, CBE094J04, CBE066B03, CBE040L24, CBE183I08, CME008L23 and CME005H15, to the *C. farreri* chromosomes by BAC-FISH [21]. These clones should be mapped to a single site of the genome if they are assembled into a single contig. Third, we screened the scallop BAC libraries by PCR using the primers designed from six genes of the *lectin* family, and checked their positions on the physical map. Finally, we selected 20 BESs from 20 contigs and designed a pair of primers from a BES contained in each of the contigs. PCR was conducted in all clones contained in each of the contigs, respectively. If the contigs are assembled properly, at least two clones of each contig should yield amplicons.

### Initial integration of the physical map with the genetic map

To facilitate the integration of the physical map with the scallop genetic linkage maps and sequencing of the scallop genome, we randomly selected BAC clones from the source BAC libraries of the physical map and sequenced their ends using the BigDye Terminator V3.1 Cycle Sequencing Kit (Applied Biosystems, Foster City, CA, USA) with the ABI 3130xl Genetic Analyzer. After trimming and quality filtering the sequences by Phred, a Parser program (developed by Texas A&M University, unpublished) was used to anchor the BAC end sequences (BESs) to the physical map. The BESs were also blasted against the NCBI non-redundant protein database (Nr), nucleotide database (Nt) and EST database [http://www.ncbi.nlm.nih.gov] of *C*. *farreri*. The annotated genes and simple sequence repeats (SSRs) were identified and used as anchors to map the contigs of physical map to the existing scallop genetic maps.

## Results

### BAC fingerprinting

**Figure 1:**
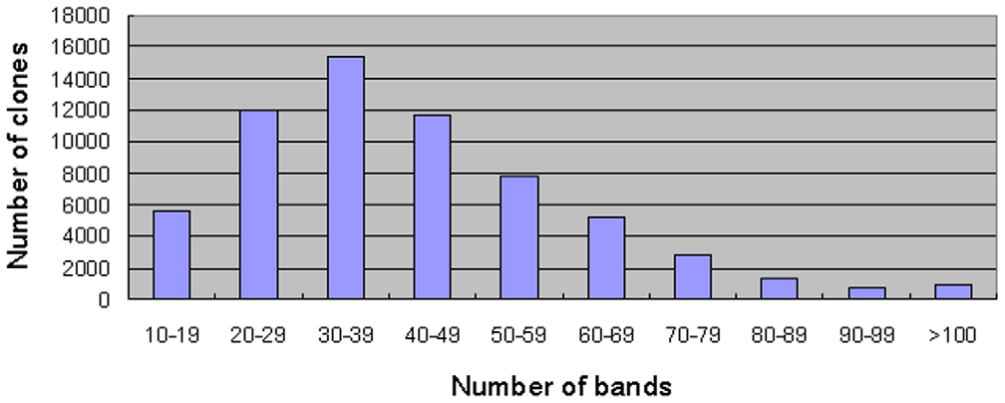
Distribution of band numbers per scallop BAC fingerprint. Most of the clones have a fingerprint consisting of 20 to 59 bands, with an average number of 41.2 bands.

A total of 81,408 (7.4× genome coverage) BAC clones were fingerprinted from the two scallop BAC libraries. Of these clones, the fingerprints of 63,641 (78.2% success) were validated and used in the physical map assembly (Table 1). The clones with validated fingerprints represented approximately 5.8 fold of the scallop haploid genome. Each BAC fingerprint consisted of an average of 41.2 restriction fragments in the window between 35 and 500 bases, with a range from 20 to 59 bands for the fingerprints of most of the BAC clones (Figure 1).

**Table 1:** BAC libraries used in the physical map construction of the scallop genome.

Libraries	Cloning site	Vector	Average Insert size ( kb)	No. of clones fingerprinted	No. of clones used in mapping	Average no. of bands/clone	Genome coverage
Scallop-CBE	*Bam*H I	pECBAC1	110	73,728	57,334	50.6	5.1×
Scallop-CME	*Mbo* I	pECBAC1	145	7,680	6,307	40.2	0.7×
Total			113	81,408	63,641	41.2	5.8×

### Determination of tolerance and cutoff values for physical map assembly

Tolerance and cutoff are the two most important parameters used in the FPC program for contig map assembly. The tolerance dictates how closely two restriction fragments in different clones must match to be considered as the same band. The tolerance value was determined in this study by the mean size deviation of the vector pECBAC1 fragment analysis (Figure S1). At a confidence level of ≥95%, the mean deviations of the three vector fragments (161 bases, 230 bases, 375 bases) were 0.297, 0.468 and 0.585 bases, respectively (Figure S1), with an average of 0.450 bases. Since all fragment sizes of the fingerprints were multiplied by 10, the tolerance value of 5 should be used for the physical map assembly. At the same time, we also tested a series of tolerances (tolerance 1–7) to determine its optimal value for contig assembly. On the basis of the test results, a tolerance of 5 was selected for the contigs assembly.

**Figure 2:**
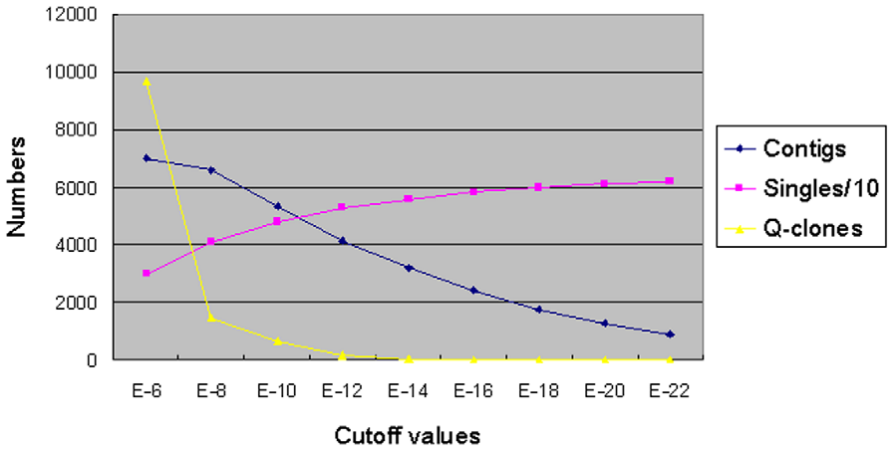
Plot of the numbers of contigs, singletons, and Q-clones versus the cutoff values used for the contig assemblies.

The cutoff value is a threshold of the probability that fingerprint bands of two clones match by coincidence. Lowering the cutoff value would increase the stringency and therefore, increase the likelihood that reported overlapping BAC clones are truly overlapping. Before we assembled the scallop physical map, we tested a series of cutoff values ranging from 1e-6 to 1e-22 for automatic contig assembly. The numbers of resultant contigs, singletons and questionable clones (Q-clones or Qs), physical map total length, and contig average length were considered. At higher stringencies or lower cutoff values (1e-14 to 1e-22), "chimeric" contigs were split, but the number of singletons increased drastically. At lower stringencies or higher cutoff values (1e-6 to 1e-8), a lower number of contigs were assembled, but a larger number of clones were in the category of Q-clones and a larger number of contigs are "chimeric". Therefore, we plotted the numbers of contigs, singletons and Q-clones versus the physical map assembly stringencies. The analysis showed that a cutoff value of approximately 1e-12 resulted in a reasonable low number of all three parameters, contigs, singletons, and Q-clones (Figure 2). Based on the test results, we, hence, chose 1e-12 as the cutoff value for assembling the physical map.

### Contig assembly and analysis

A total of 3,696 contigs were assembled with the validated fingerprints of 63,641 BACs using the FPC program with a tolerance of 5 and a cutoff value of 1e-12, followed by end-to-end, end-to-single merging manually and Dqer processing. A total of 37,046 clones were assembled into the contigs, whereas the remaining 26,595 clones remained as singletons.

**Figure 3:**
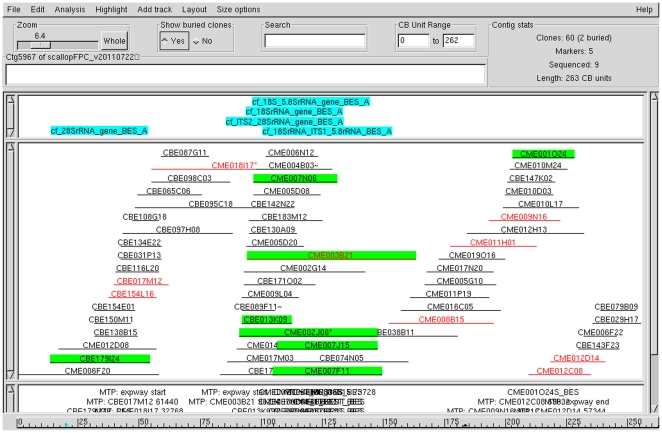
A contig of the scallop physical map. The Ctg5967 consists of 60 clones spanning 262 CB units equivalent to 1,077 kb in physical length. The eight highlighted clones are the positive clones of the markers containing 18S-28S rRNA gene.

The statistics of the physical map contigs is shown in Table 2. Most of the contigs (95.5%) contained 3–24 BAC clones, which contributed to 94.0% of the consensus band (CB) units of the physical map. The contig containing the largest number of clones consisted of 60 clones, spanning a physical length of 1,077 kb (Figure 3); the largest contig contained 46 clones, covering 1,683 kb in physical length; and the smallest contig contained 2 clones, spanning a physical length of 65 kb. The contigs had an average number clone of 10.0 and an average physical length of 490 kb.

**Table 2:** Statistics of the scallop BAC physical map.

Total number of BAC clones fingerprinted	81,408	7.4× genome coverage
Validated fingerprints for map assembly	63,641	5.8× genome coverage
Total number of contigs assembled	3,696	
Contigs containing 50–99 clones	1	
Contigs containing 25–49 clones	105	
Contigs containing 10–24 clones	1,606	
Contigs containing 3–9 clones	1,925	
Contigs containing 2 clones	59	
Clones contained in the 3,696 contigs	37,046	
Average BAC clones per contig	10.0	
Average estimated size per contig (kb)	490	
N50 of contig size (kb)	458	
Total number of CBs included in the contigs	442,884	
Number of singletons	26,595	
BESs in the contigs	10,587	
BESs in the singletons	4,792	
Markers in the contigs	167	
Total physical length of assembled contigs (kb)	1,812,223	∼1.5× genome size

There were a total of 442,884 CBs distributed in the 3,696 contigs of the physical map. This represents approximately 1.81 Gb physical length of DNA, with an average CB size of 4.092 kb (1,812,223 kb/442,884 CBs). The 1.81-Gb physical length of the physical map contigs was equivalent to approximately 1.5 fold of the Zhikong scallop genome size (Table 2). Each BAC in the contigs contributed an average of 12.0 unique CBs to the physical map, or approximately 49.1 kb to the physical length of the contig map. In addition, we picked a total of 9,130 MTP clones, spanning a total of 1,505,055 kb, using the MTP (minimal tiling path) module of the FPC program. The contigs files can be viewed in www.crustacea.org.cn/7th/upload/PLoS_ONE/scallopFPC_v20110722.rar.

### Assessment of the physical map reliability

To assess the quality of the physical map, the 190 BAC clones constituting 18 contigs randomly selected from the scallop physical map were fingerprinted with a new restriction enzyme combination consisting of *Bam*H I/*Eco*R I/*Xho* I/*Hae* III, and re-assembled into contigs. A total of 180 of the clones were re-assembled into 21 contigs (Table S1). Comparing the reassembled contigs with the original contigs showed that 11 of the 18 original contigs selected from the scallop physical map completely matched with reassembled contigs, 4 each lost one or two clones, and the remaining 3 original contigs were each split into two contigs in the reassembly. This result indicated that the contigs of the scallop physical map could be reassembled using different fingerprinting methods, suggesting that they were assembled properly.

Next, the BAC clones containing six genes involved in the mollusc innate immune system identified in our previous study [1] were used to validate the contig map assembly (Table 3). We first checked the positions of the positive clones of the genes in the physical map. The positive clones of each of four, *hsp70*, *lgbp*, *ndpk*, *serine protease*, of the six genes analyzed were located to a single contig, confirming the accuracy of the contig assembly. The positive clones of the remaining two genes, *serine protease inhibitor* and *hemocyanin*, were located to two or more contigs, respectively. This result could be attributed to the multi-copies of the genes, but could not exclude the possibility of improper assembly (Table 3). It is also possible that the contigs containing the positive clones of each of the genes could be merged, if additional clones and markers are analyzed. Further, the six positive clones of the *lgbp* gene, CBE094J04, CBE066B03, CBE040L24, CBE183I08, CME008L23 and CME005H15, were mapped to the scallop chromosomes by double-color FISH using CBE094J04 as the reference marker (Figure S2). It was found that CBE094J04 was co-localized with all of the five remaining clones [21]. This result further confirmed the accuracy of the contig assembly.

**Table 3:** Locations of positive BACs of six genes involved in the innate immune system of mollusc.

Genes	Number of positive clones	Contig	Name of positive clones
*hsp 70*	3	Ctg6406	**CBE040F20**
		Ctg1982	**CBE123C08,CME011M12**
*lgbp*	6	Ctg7640	**CBE040L24,CBE066B03,CBE094J04, CBE183I08,CME005H15,CME008L23**
*ndpk*	2	Ctg6055	**CBE068E24,CBE099F20**
*Serine protease*	6	Ctg6406	**CBE024L03,CBE032A07,CBE040H03, CME017A05,CME017B14**
		Singleton	CBE109E21
*Serine protease inhibitor*	4	Ctg59	**CBE126O09**
		Ctg6052	**CME013J06**
		Singleton	CBE027F23
		Fingerprint failed	C*BE191H2*
*hemocyanin*	23	Ctg117	**CBE075D21**
		Ctg1097	**CBE182C08**
		Ctg2480	**CBE157I24, CBE187A08**
		Ctg6118	**CBE065L13, CBE071P16, CBE108A09**
		Ctg6120	**CBE104C21, CBE185C08**
		Ctg6886	**CBE024P13, CBE165O11, CBE166O15**
		Ctg7623	**CBE181C24**
		Ctg7809	**CBE143D23, CBE156F16,CBE169I03,**
		Singleton	CBE025I03, CBE067N18, CBE107C24, CBE174F22, CBE187E22, CME008P07
		Fingerprint failed	*CBE145B23*

Hybridization was conducted using the high-density filters of the scallop libraries [1]. Boldface indicates fingerprinted clones mapped in the physical map and normal font indicates fingerprinted clones in singleton. Clones in italic font failed in fingerprinting.

Third, we screened the source BAC libraries of the physical map by PCR using the primers designed from six genes of the *lectin* family. A total of 20 positive BACs were obtained. At least two positive clones of each of four of the six genes were located to a single contig, and the gene *lectin 6* had its all five positive clones located to one contig (Ctg2936).

Finally, we analyzed all of the 212 BACs contained in 20 contigs randomly selected from the physical map using PCR. As shown in Table S2, every contig analyzed was shown to have at least two positive BAC clones identified by PCR. These results have provided another line of evidence for the reliability of the physical map.

### Initial integration of the physical map with the scallop genetic map

**Figure 4:**
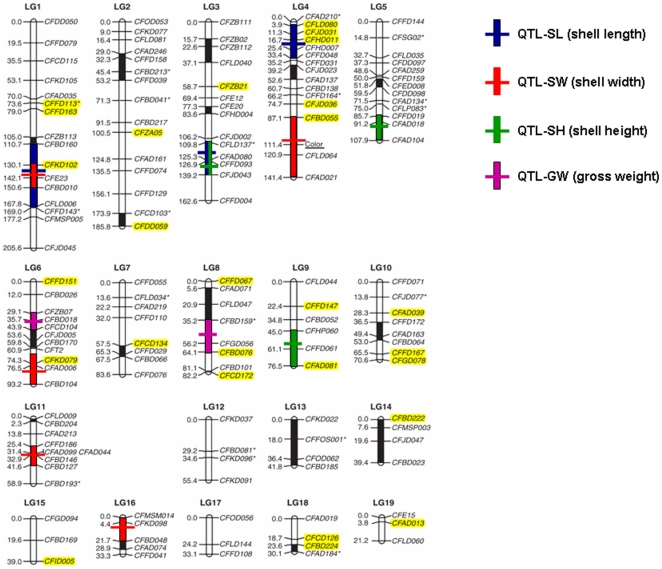
Initial integration of the scallop physical map with the scallop microsatellite linkage map. The SSR markers highlighted in yellow represent the contigs of the scallop physical map anchored with the markers. The linkage map was modified from [7].

Furthermore, we generated 17,447 BESs from the BACs randomly selected from the source BAC libraries of the physical map to facilitate its integration with the scallop genetic map and large-scale genome sequencing. The BESs were submitted to GenBank with accession numbers of JM408914-JM426360. From the BESs, A total of 15,379 BESs were mapped to the physical map, with 10,587 in the map contigs and 4,792 in singletons. Approximately 68% of the contigs have at least one BES marker. To distinguish from the other type of DNA markers, the BES markers were defined as remarkers on the physical map. By BLAST against the Nr, Nt and EST databases, 408 of the BESs were annotated into 28 scallop functional genes and 1,482 had significant hits to 107 sequences containing *C. farreri* microsatellite, thus providing new markers to anchor the physical map contigs to a genetic map. Among the 107 microsatellite markers, 27 were located to 13 linkage groups of the existing microsatellite linkage map of the scallop genome [7]; therefore, their containing contigs were anchored to the scallop genetic map. In the 27 markers, 8 were closely linked with scallop size-related QTLs: CFKD102 (QTL-SL, QTL-SW), CFLD080 (QTL-SL), CFJD031 (QTL-SL), CFHD011 (QTL-SL), CFBD055 (QTL-SW), CFKD079 (QTL-SW), CFAD081 (QTL-SH), and CFBD076 (QTL-GW) (Figure 4).

## Discussion

In this study, we have fingerprinted 81,408 (7.4-fold genome) BACs from two BAC libraries, and developed a genome-wide BAC physical map of scallop. The physical map consists of 3,696 contigs, with a contig physical size range 65 kb to 1,683 kb and an average physical length of 490 kb per contig. The average contig size of the physical map is well comparable to those that have been reported [13]–[16], [22], [24]–[31]. The physical map contigs collectively span approximately 1.81 Gb, equivalent to approximately 1.5 fold of the scallop haploid genome. The larger total length of the contigs than the scallop genome size could be attributed to either the underestimation of the scallop genome size or the existence of extensive overlaps between contigs. Nevertheless, it appears that the latter plays a much more important role in the regard because the FPC program used for the physical map assembly only can assemble the clones that are significantly overlapped into contigs or merge the contigs that are significantly overlapped (http://www.agcol.arizona.edu/software/fpc/). Moreover, we have integrated 15,379 BESs and 167 markers, including 40 genes, into the physical map (Table 4). Such a BAC-based physical map is significant for advanced research of genomics and genetics in the scallop and related mollusc species. These include, but are not limited to, large-scale genome sequencing using the modern NGS technology, map-based cloning of genes and QTL of economical importance, genetic improvement and selective breeding.

**Table 4:** List of all markers on the *C. farreri* physical map.

Origin	Number	Name of markers
Overgo hybridization	6	hemocyanin, hsp70, LGBP, NDPK, serine_protease, serine_protease_inhibito*r*
BACs screening using PCR primers designed from contig BESs	20	CBE001M07_PCR, CBE003L17_PCR, CBE003O12_PCR, CBE003P01_PCR, CBE003P17_PCR, CBE004E17_PCR, CBE004I09_PCR, CBE004I17_PCR, CBE012A15_PCR, CBE012C03_PCR, CBE012C11_PCR, CBE012E07_PCR, CBE012I05_PCR, CBE013A09_PCR, CBE013E17_PCR, CBE013H01_PCR, CBE013M02_PCR, CBE013M05_PCR, CBE013M13_PCR, CBE013O09_PCR
BESs hit microsatellite locus sequences	107	CFAD004_MS, **CFAD013_MS(LG19, Ctg6249)**, CFAD026_MS, **CFAD039_MS(LG10, Ctg3076), CFAD081_MS(LG9, Ctg7094),** CFAD091_MS, CFAD101_MS, CFAD119_MS, CFAD121_MS, CFAD145_MS, CFAD166_MS, CFAD170_MS, CFAD172_MS, CFAD174_MS, FAD175_MS, CFAD191_MS, CFAD243_MS, CFAH_MS, CFAI_MS, CFBD008_MS, CFBD012_MS, **CFBD055_MS(LG4, Ctg6587), CFBD076_MS(LG8, Ctg7491, Ctg7087, Ctg1308),** CFBD100_MS, CFBD103_MS, CFBD120_MS, CFBD122_MS, CFBD128_MS, CFBD129_MS, CFBD136_MS, CFBD160_MS, CFBD163_MS, CFBD164_MS, CFBD165_MS, CFBD177_MS, CFBD178_MS, CFBD186_MS, CFBD187_MS, CFBD194_MS, CFBD196_MS, CFBD200_MS, **CFBD222_MS(LG14, Ctg632, Ctg3081, Ctg7382, Ctg7320), CFBD224_MS(LG18, Ctg592),** CFCD103_MS, **CFCD126_MS(LG18, Ctg 6079), CFCD134_MS(LG7, Ctg7336),** CFCD140_MS, **CFCD172_MS(LG8, Ctg2444), CFDD059_MS(LG2, Ctg7607, Ctg6127),** CFDD068_MS, CFFD032_MS, CFFD036_MS, CFFD047_MS, CFFD050_MS, CFFD057_MS, CFFD065_MS, **CFFD067_MS(LG8, Ctg6367),** CFFD081_MS, CFFD107_MS, **CFFD113_MS(LG1, Ctg164),** CFFD114_MS, CFFD122_MS, CFFD124_MS, CFFD125_MS, CFFD127_MS, CFFD143_MS, CFFD146_MS, **CFFD147_MS(LG9, Ctg4352), CFFD151_MS(LG6, Ctg2897),** CFFD156_MS, CFFD161_MS, **CFFD163_MS(LG1, Ctg7666, Ctg5889, Ctg1872),** CFFD165_MS, **CFFD167_MS(LG10, Ctg7552),** CFFD196_MS, **CFGD078_MS(LG10, Ctg972, Ctg6835), CFHD011_MS(LG4, Ctg6416), CFID005_MS(LG15, Ctg7676), CFJD031_MS(LG4, Ctg3076), CFJD036_MS(LG4, Ctg3833), CFKD079_MS(LG6, Ctg188), CFKD102_MS(LG1, Ctg6880),** CFKD113_MS, CFLD080_MS(LG4, Ctg6367), CFLD094_MS, CFLD095_MS, CFLD133_MS, CFML06_MS, CFMSM007_MS, CfP009_MS, CfP929_MS, CFSG02_MS, CFSSR0065_MS, CFSSRC004_MS, CFSSRC005_MS, CFSSRD002_MS, CFSSRF041_MS, CFSSRK070_MS, **CFZA05_MS(LG2, Ctg6367),** CFZB10_MS, **CFZB21_MS(LG3, Ctg3596),** CFZB41_MS, CFZB89_MS, CFZB96_MS, CFZM015_MS, iolscf0_006511_MS, iolscf0_007259_MS
BESs hit genes	28	cf_myostatin_gene_BES_A, cf_TEP_gene_BES_A, cf_5SrRNA_gene_NTS_BES_A, cf_18SrRNA_gene_BES_A, cf_18SrRNA_ITS1_5.8rRNA_BES_A, cf_18S_5.8SrRNA_gene_BES_A, cf_28SrRNA_gene_BES_A, cf_AhR_gene_T_BES_A, cf_beta_tubulin_gene_S_BES_A, cf_catalase_T_BES_A, cf_cathepsinD_gene_T_BES_A, cf_cytP450_4_gene_T_BES_A, cf_HistoneH2A_T_BES_A, cf_histone_gene_cluster_BES_A, cf_hsp70_gene_BES_A, cf_ITS2_28SrRNA_gene_BES_A, cf_LGBP_S_BES_A, cf_polyprotein_gene_BES_A, cf_sp_inhibitor_gene_BES_A, cf_TNFreceptor_pre_gene_BES_A, cf_TRAF6_T_BES_A, cf_lectin6_BES_A, cf_tRNA(Gul)_ BES, cf_tRNA(Thr)_BES, cf_tRNA(Ser)_BES, cf_tRNA(Pro)_BES, cf_tRNA(Leu)_BES, cf_tRNA(Cys)_BES
Library screening by gene primers	6	cf_lectin1_PS, cf_lectin2_PS, cf_lectin3_PS, cf_lectin4_PS, cf_lectin5_PS, cf_lectin6_PS
BESs (as remarker)	10,587	www.crustacea.org.cn/7th/upload/PLoS_ONE/scallopFPC_v20110722.rar

The markers highlighted in boldface are the SSRs mapped on the genetic linkage map of *C. farreri* [7]. The “LGx” in parentheses are the names of linkage groups of the map, The “Ctgx” is the contig name of the *C. farreri* physical map.

The scallop physical map has a reasonable genome coverage and the contigs constituting the map are assembled properly. The physical map was assembled from the validated fingerprints of 63,641 clones, equivalent to 5.8 fold of the scallop haploid genome. This genome coverage of clones is similar or larger than those used for the physical maps of Nile tilapia (5.58×) [11], catfish (5.6×) [13], Asian seabass (4.5×) [16], peach (4.3×) [24] and melon (5.7×) [25]. Moreover, the fingerprinted clones of the scallop physical map were constructed with two restriction enzymes (*Bam*H I and *Mbo* I), respectively, which further enhances the map's representation for the scallop genome [32]. Furthermore, the physical map has been verified using several methods, including contig clone fingerprinting using new fingerprinting kits and contig reassembly, BAC-FISH, library screening, and contig BAC screening. The results of the analyses consistently suggest that the physical map contigs have been assembled properly.

Two fingerprinting methods have been used for physical mapping with BACs [13]–[16], [22], [24]–[31]. One method digests a BAC DNA with 1–4 restriction enzymes and labels the restricted fragments with one fluorescent dye, usually yielding an average number of 30–70 bands per BAC fingerprint [30], [31]. The other method is the High-Information Content Fingerprinting (HICF) method. A BAC DNA is digested with five restriction enzymes (*Hin*d III, *Eco*R I, *Xba* I, *Xho* I and *Hea* III) and labeled using the SNaPshot kit (Applied Biosystems, USA) containing four fluorescent dyes (R110/R6G/TAMRA/ROX) [29], [31]. In this study, we have developed and used a new fingerprinting method in which a BAC DNA is digested with 4 restriction enzymes (*Hin*d III, *Xba* I, *Xho* I and *Hea* III), and labeled with one fluorescent dye (carboxyrhodamine fluorescent dye) in one-step one-tube reaction. Since fewer restriction enzymes and one fluorescent labeling dye are used in our method, the problems that often occur in one-step one-tube reaction in the HICF method, such as partial digestion, star activity and low labeling efficiency [29], are minimized. Moreover, the one-color labeling system of the new method allows selection of restriction enzyme combination that is optimal for fingerprinting BACs of a species for quality physical map construction. Furthermore and importantly, previous studies showed that the fingerprinting BACs using one color labeling system usually results in physical maps with much larger average contig sizes (e.g., Nile tilapia, 484 kb; chickpea, 559 kb; chicken, 648 kb; soybean, 485 kb) [11], [22], [30], [31] than that with the SNaPshot multiple-color labeling system (e.g., Catfish, 292 kb; rainbow trout, 130 kb; Asian seabass, 232 kb; peach, 142 kb; melon, 300 kb) [13], [15], [16], [24], [25]. Finally, the new one-color labeling system is several-fold more economical than the SNaPshot labeling system because inexpensive fluorescent dye and conventional Taq DNA Polymerase are used in the system. Therefore, the fingerprinting method developed in this study is widely applicable for genome physical mapping of different species with BACs.

The scallop physical map will greatly promote advanced research of the scallop genome in many aspects. First, fifteen contigs containing *hsp70*, *LGBP*, *NDPK*, *serine protease*, *serine protease inhibitor* and *hemocyanin* that are involved in the scallop innate immune system have been identified from the physical map. These contigs provide useful tools and resources to further characterize the genes and determine their roles in resistance to pathogens. Currently, there are 3,537 ESTs of *C. farreri* in the NCBI (as of August, 2011). It is believed that a much larger number of ESTs containing genes of interest will be generated soon using the next generation RNA-sequencing technology. Availability of the scallop physical map will greatly promote use of the EST resources to clone and characterize genes and QTL important to aquaculture. Using physical maps, chromosomal regional markers can be developed from targeted genomic regions for fine mapping of candidate genes associated with traits important to aquaculture. Second, we have generated in this study over 15,000 BESs from the source BACs of the physical map and using them as a tool, mapped 27 contigs of the physical map to 13 linkage groups of the scallop genetic linkage map. This experiment has not only provided a large number of sequence-tagged sites (STSs) along the scallop genome, but also demonstrated the feasibility and provided tools of using the BESs to integrate the physical map with the existing genetic map of the species [7]. It has been shown that integrated BAC physical/genetic maps are essential for many advanced genome research endeavors, e.g., whole-genome sequencing, high-resolution high-throughput genome mapping, large-scale genome functional analysis and gene/QTL cloning [33]. Finally, an international collaboration project will be launched soon to sequence the *C. farreri* genome using the next-generation sequencing technology. The BAC-based scallop physical map developed in this study has provided a useful framework to assemble and refine the genome sequence, or will become a foundation for sequencing the scallop genome. It has been shown that pool sequencing of minimal tilling path (MTP) BACs of physical maps is a promising approach to *de novo* sequencing of large and complex genomes [9].

### Conclusion

We have developed a quality BAC-based physical map of the scallop genome. This physical map represents the first physical map of the species. Because such physical maps have been demonstrated in many species to be essential for many advanced studies of genomics, genetics and breeding, the scallop physical map that we developed in this study will greatly promote research of genomics, genetics and breeding of the species in many aspects, particularly cloning and characterization of genes and QTLs important to aquaculture, genome sequencing and assembly, large-scale genome comparative analysis with related species such as bivalves and molluscs. The fingerprinting method developed and used in the construction of the physical map will be applicable to the construction of physical maps of different species due to its high reproducibility, high flexibility and high cost efficiency.

## Supporting Information

Figure S1 **The size distributions of three vector fragments. **The tolerance value used for the physical map assembly was determined by the mean size deviation of the vector pECBAC1 fragments derived from fingerprints peaks. With a confidence level of ≥95%, the mean deviations of the three vector fragments (161 bases, 230 bases and 375 bases) were 0.297, 0.468 and 0.585 bases, respectively, with an average of 0.450 bases. **a**, 161-base fragment; **b**, 230-base fragment; **c,** 375-base fragment.
(PPT)

Figure S2 **Double-color FISH showing the six** ***lgbp*** **-containing BACs co-localized at the same site of the** ***C. farreri*** **genome. **The green signals indicate the localization of the clone CBE094J04, the red signals of each set indicate the locations of the remaining five clones, respectively. The signals are indicated by arrows [21].
(PPT)

Table S1 **Reassembling BAC contigs for assessment of the scallop physical map. **One hundred ninety BACs constituting 18 contigs randomly selected from the scallop physical map were fingerprinted with *Bam*H I/*Eco*R I/*Xho* I/*Hae* III, and re-assembled into contigs. A total of 180 of the clones were assembled into 21 contigs. Eleven of the 18 original contigs completely matched with reassembled contigs, three were each split into two contigs, and the remaining four each lost one or two clones. The attached figure shows an original contig of the *C. farreri* physical map (A) and its reassembly (B).
(DOC)

Table S2 **Assessing the scallop physical map reliability by contig BAC screening using PCR. **A total of 212 BAC clones of 20 contigs randomly selected from the scallop physical map were analyzed by PCR using the primers designed from the BESs of the contigs. If the BACs of a contig are assembled properly, at least two of them should be identified using a pair of primers designed from the BES of one of its clones. It was found that every contig analyzed has at least two BACs yielding amplicons, thus providing a line of evidence for the reliability of the physical map.
(DOC)
