# Supplementary material for: A BAC-Based Physical Map of Zhikong Scallop (Chlamys farreri Jones et Preston)
Source: PLoS One. 2011 Nov 16;6(11):e27612. doi: 10.1371/journal.pone.0027612 (PMC3218002; doi:10.1371/journal.pone.0027612)
Supplement: Figure S1 — The size distributions of three vector fragments. The tolerance value used for the physical map assembly was determined by the mean size deviation of the vector pECBAC1 fragments derived from fingerprints peaks. With a confidence level of ≥95%, the mean deviations of the three vector fragments (161 bases, 230 bases and 375 bases) were 0.297, 0.468 and 0.585 bases, respectively, with an average of 0.450 bases. a, 161-base fragment; b, 230-base fragment; c, 375-base fragment. (PPT) [file pone.0027612.s001.ppt]

## Slide 1
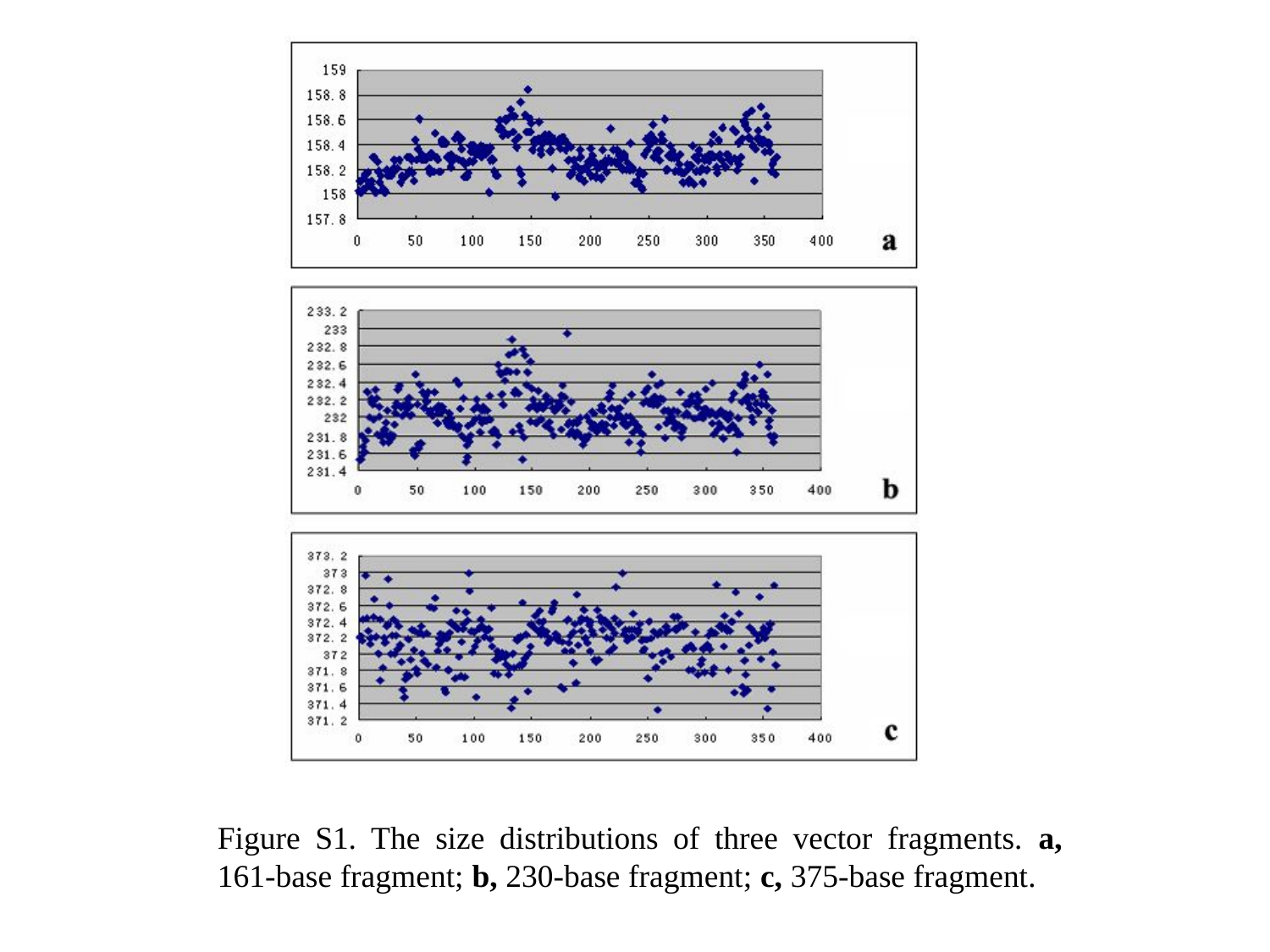

# Figure S1. The size distributions of three vector fragments. a, 161-base fragment; b, 230-base fragment; c, 375-base fragment.
