# Supplementary material for: A BAC-Based Physical Map of Zhikong Scallop (Chlamys farreri Jones et Preston)
Source: PLoS One. 2011 Nov 16;6(11):e27612. doi: 10.1371/journal.pone.0027612 (PMC3218002; doi:10.1371/journal.pone.0027612)
Supplement: Figure S2 — Double-color FISH showing the six lgbp -containing BACs co-localized at the same site of the C. farreri genome. The green signals indicate the localization of the clone CBE094J04, the red signals of each set indicate the locations of the remaining five clones, respectively. The signals are indicated by arrows [21]. (PPT) [file pone.0027612.s002.ppt]

## Slide 1
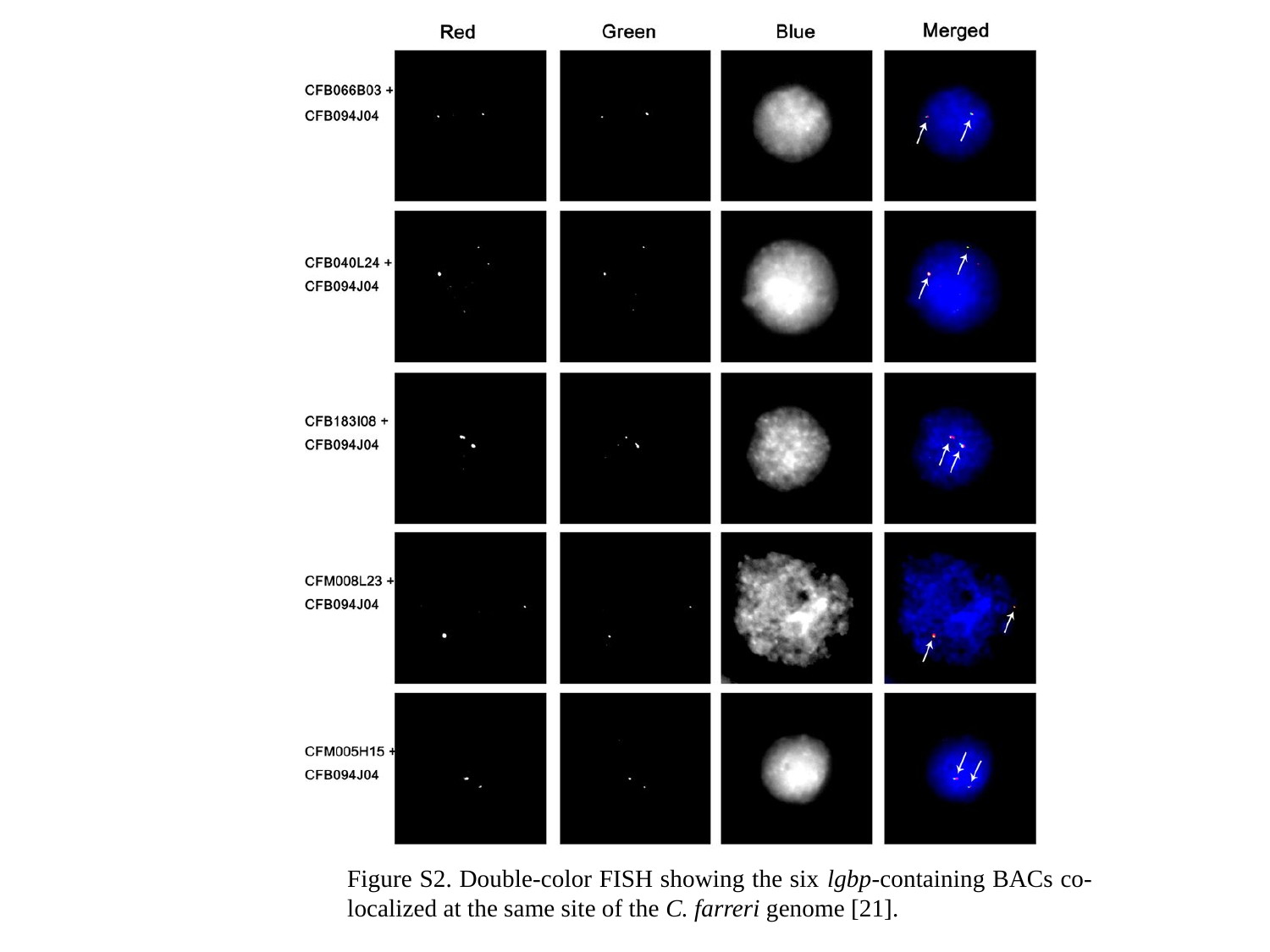

Figure S2. Double-color FISH showing the six lgbp-containing BACs co-localized at the same site of the C. farreri genome [21].
