# Supplementary material for: A BAC-Based Physical Map of Zhikong Scallop (Chlamys farreri Jones et Preston)
Source: PLoS One. 2011 Nov 16;6(11):e27612. doi: 10.1371/journal.pone.0027612 (PMC3218002; doi:10.1371/journal.pone.0027612)
Supplement: Table S1 — Reassembling BAC contigs for assessment of the scallop physical map. One hundred ninety BACs constituting 18 contigs randomly selected from the scallop physical map were fingerprinted with BamH I/EcoR I/Xho I/Hae III, and re-assembled into contigs. A total of 180 of the clones were assembled into 21 contigs. Eleven of the 18 original contigs completely matched with reassembled contigs, three were each split into two contigs, and the remaining four each lost one or two clones. The attached figure shows an original contig of the C. farreri physical map (A) and its reassembly (B). (DOC) [file pone.0027612.s003.doc]

**Table S1. Reassembling BAC contigs for assessment of the scallop physical map.**

| Name of original contig in the physical map | No. of clones | Name of contig in the assembly | No. of clones | Percentage  of match |
| --- | --- | --- | --- | --- |
| Ctg130 | 16 | Ctg15 | 9 | Split |
|  |  | Ctg18 | 7 |
| Ctg258 | 16 | Ctg7 | 12 | Split |
|  |  | Ctg20 | 4 |
| Ctg467 | 7 | Ctg24 | 6 | 85.7% |
| Ctg802 | 9 | Ctg22 | 8 | 88.9% |
| Ctg1090 | 6 | Ctg32 | 6 | 100% |
| Ctg1211 | 4 | Ctg29 | 4 | 100% |
| Ctg1633 | 15 | Ctg13 | 15 | 100% |
| Ctg2453 | 5 | Ctg16 | 2 | 100% |
| Ctg2543 | 5 | Ctg12 | 5 | 100% |
| Ctg3175 | 13 | Ctg5 | 13 | 100% |
| Ctg3953 | 13 | Ctg31 | 11 | 84.6% |
| Ctg4134 | 3 | Ctg25 | 2 | 100% |
| Ctg4727 | 13 | Ctg33 | 13 | 100% |
| Ctg4962 | 14 | Ctg10 | 14 | 100% |
| Ctg5050 | 25 | Ctg2 | 9 | Split |
|  |  | Ctg3 | 16 |
| Ctg6410 | 10 | Ctg28 | 8 | 80.0 % |
| Ctg6720 | 6 | Ctg17 | 6 | 100% |
| Ctg6896 | 10 | Ctg4 | 10 | 100% |


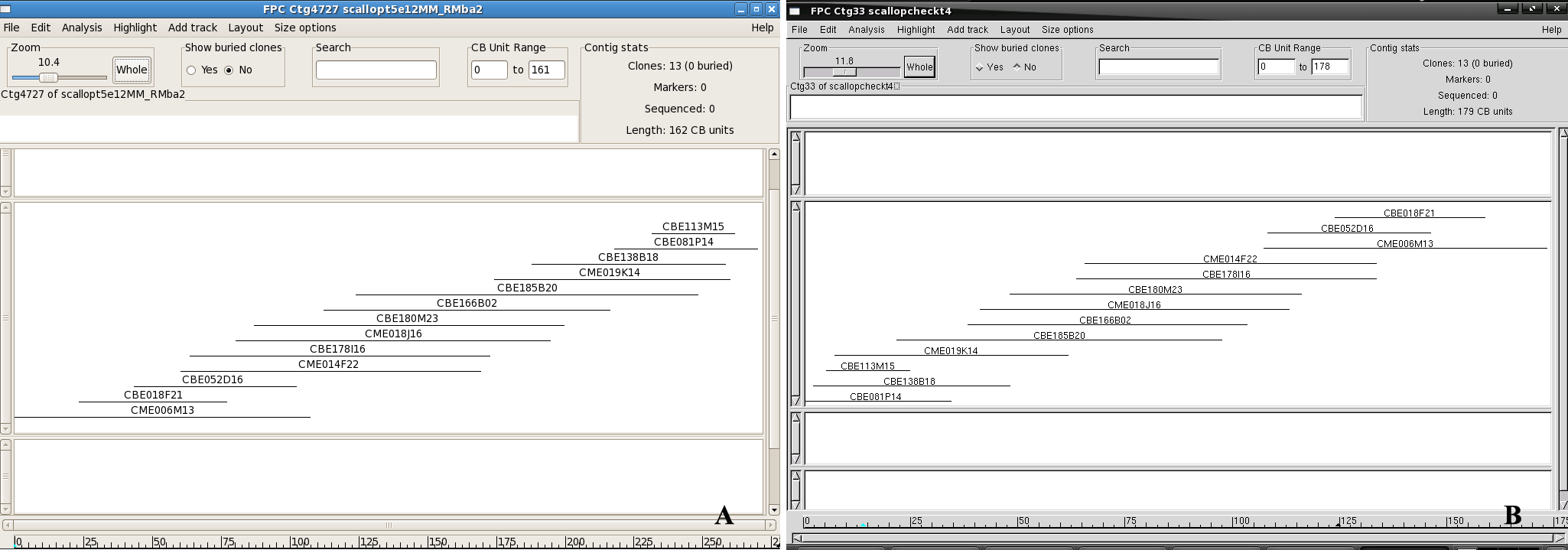


**An original contig of the scallop physical map (A) and its reassembly (B)**
