# Supplementary material for: A BAC-Based Physical Map of Zhikong Scallop (Chlamys farreri Jones et Preston)
Source: PLoS One. 2011 Nov 16;6(11):e27612. doi: 10.1371/journal.pone.0027612 (PMC3218002; doi:10.1371/journal.pone.0027612)
Supplement: Table S2 — Assessing the scallop physical map reliability by contig BAC screening using PCR. A total of 212 BAC clones of 20 contigs randomly selected from the scallop physical map were analyzed by PCR using the primers designed from the BESs of the contigs. If the BACs of a contig are assembled properly, at least two of them should be identified using a pair of primers designed from the BES of one of its clones. It was found that every contig analyzed has at least two BACs yielding amplicons, thus providing a line of evidence for the reliability of the physical map. (DOC) [file pone.0027612.s004.doc]

**Table S2.**  **Assessing the physical map reliability by Contig BAC screening using PCR**

| Contig screened | No. of clones | Name of the clones | Source BAC of PCR primers | No. of positive clones |
| --- | --- | --- | --- | --- |
| Ctg4 | 5 | CBE161J23, CBE161J23**, CBE174N01**, **CBE013M13**, **CBE143C09** | CBE013M13 | 3 |
| Ctg372 | 11 | CME003L09, CBE147L20, **CBE166D06, CME005G11, CBE157J06,** CBE074E20, **CBE003P17,** CBE185G09, CBE087L21, CBE123G03, CBE171M01 | CBE003P17 | 4 |
| Ctg467 | 7 | CBE176G08, CBE166G22, **CME017M24**, **CBE003L17**, CBE129M02, CBE124I01, **CBE139B21** | CBE003L17 | 3 |
| Ctg707 | 9 | **CME018C14**, CBE089P23, **CBE181H15**, **CBE013O09**, **CBE033H10, CME016P09, CBE036A10,** CBE182I05, CBE098J05 | CBE013O09 | 6 |
| Ctg802 | 9 | CME008M16, CBE154B19, CBE165G19, **CME020C22**, CBE120G22, CBE021L03, **CBE012A15**, CBE152G20, **CBE026M24**, | CBE012A15 | 3 |
| Ctg1090 | 6 | **CBE003P01**, **CBE030D08**, CBE056F20, CBE031B20, CBE109I12, CBE059K06 | CBE003P01 | 2 |
| Ctg1211 | 4 | **CBE161O04**, **CBE012C03**, **CBE133C23**, CBE065B12 | CBE012C03 | 3 |
| Ctg1968 | 6 | **CBE120C06**, **CBE013H01**, CBE146N07, CBE158M02, CBE149F15, CBE004D23 | CBE013H01 | 2 |
| Ctg3175 | 13 | CBE109F14, CBE061G09, CBE010H17, CBE168O23, CME015N20, CBE092I12, **CBE155O12,** CBE093F21, **CBE149O24, CBE003O12, CBE151H11, CBE022L13**, CBE010N14 | CBE003O12 | 5 |
| Ctg3953 | 13 | CBE188P02, CBE180A16, **CBE058F03**, **CBE158F08, CBE043O03**, **CBE089G19,** **CBE086F07**, CBE116M24, CBE040F06, CBE017N14**,** CBE182N22, CBE006P19, CBE055J04 | CBE004I17 | 5 |
| Ctg4002 | 12 | CBE080H17, **CBE185A22**, **CME011D23**, **CBE029J08**, **CBE001M07**, CBE060L23, CME005D05, CBE021H12, CME013E22, CBE034F24, CBE162M03, CBE063P13 | CBE001M07 | 4 |
| Ctg4064 | 13 | CME009H17, CBE096N04, CBE015F08, CBE065O18, **CBE160D08**, **CBE013M05,** CBE063C24**, CBE120L13, CBE140G01, CBE037H19**，CBE045A07**,** CME004L06, CME008P02 | CBE013M05 | 5 |
| Ctg4962 | 14 | CBE163E09, CBE163F10, CBE079M03, CBE026D04, CME007D04, CBE144D03, CME006L22, CME008A11, CME007D17, **CME006N20**, **CBE123D18**,  **CBE185I22**, **CBE013E17**, **CBE150H19** | CBE013E17 | 5 |
| Ctg5050 | 25 | CBE056D03, CBE039H09, CBE104K21, CBE107M13, CBE184024, CBE104H24, CBE105A22, CBE105O16, CME001J18, CBE076N05, **CBE164H20, CBE096D12,** CME015A01, **CBE169D06, CBE172M14, CBE132J11,** CBE062F05,  **CBE012C11, CME014L14**, **CBE156G13,** CBE141N07, **CBE183N06**, CBE190J08, CBE143A05, CBE130B05 | CBE012C11 | 9 |
| Ctg5214 | 12 | CME019P19, CBE124A20, CBE080J07, CBE018D08, CBE001E01, **CBE078N22**, **CBE004I09**, CBE041C13, CBE015 M01, CBE041K13, CBE018C16, CBE059D14 | CBE004I09 | 2 |
| Ctg6410 | 10 | CBE059M03, CBE146E18, CBE186B11, **CBE157H04**, **CBE161A11**, **CBE012I05,** CBE044O01, CBE138B04, CBE150EO8, CBE138A02, | CBE012I05 | 3 |
| Ctg6688 | 17 | CBE045K02, **CBE027L04, CBE111C09, CBE180P11**, **CBE141K07**，**CBE169A03, CME010A03**，CBE162A17, **CBE168B14, CBE181L15, CBE163M16**, **CBE012E07, CBE131J03, CBE021P19, CBE174C04**, **CBE056K20, CBE087K20** | CBE012E07 | 15 |
| Ctg6720 | 6 | **CME005B23**, CBE101I15, CBE109C12 , **CBE176L13**, **CBE084O03**, **CBE013A09** | CBE013A09 | 4 |
| Ctg6892 | 10 | CBE089G12, CBE156H08, CBE126J20, CME017F04, **CBE154D01**, **CME018N18**, **CBE013L02**, **CBE013M02**, CME020I22, CBE083I05 | CBE013M02 | 4 |
| Ctg6896 | 10 | CBE020F06, CBE005P19, **CBE008M11, CBE043H08, CBE146K23, CBE044O06, CBE080J16, CBE004E17, CBE109E04, CBE105D05** | CBE004E17 | 8 |

Boldface indicates the fingerprinted clones identified by PCR, and the normal font indicates the fingerprinted clones not identified by PCR.
